# Effect of whole-body cryotherapy on recovery after high-intensity training in elite rowers

**DOI:** 10.3389/fphys.2024.1428554

**Published:** 2024-08-30

**Authors:** Tao Huang, Linfei Dan, Weirui Wang, Jiarui Ren, Xin Liu, Jianshe Li

**Affiliations:** ^1^ Faculty of Sport Science, Ningbo University, Ningbo, China; ^2^ Research Academy of Grand Health, Ningbo University, Ningbo, China

**Keywords:** whole-body cryotherapy, rowers, functional recovery, high-intensity training, blood lactate

## Abstract

The purpose of this study was to investigate the effect of whole-body cryotherapy (WBC) on acute recovery after a single high-intensity training day. Twelve elite professional male rowers from the national aquatic training base. They were randomly divided into a WBC group (n = 6) and a control group (CON group, n = 6). They performed a high-intensity training program, with a single session immediately followed by WBC (−110°C, 3 min) or recovered naturally for 3 min (CON group). Rowing performance, skin temperature, heart rate, blood pressure, and blood lactate concentrations were recorded before training, immediately, 5 min, and 15 min after the intervention. Blood samples were collected early in the morning of the day of intervention and that of the following day. The results indicated that 1) the blood lactate concentrations after WBC were significantly lower than pre-training (*p* < 0.05); 2) the maximum power significantly decreased immediately after WBC compared to pre-training (*p* < 0.05); 3) a significant main effect of time was observed for average speed, which significantly decreased after WBC (*p* < 0.05); 4) a significant main effect of time for blood parameters was observed. Specifically, hematocrit, cortisol, and hemoglobin were significantly lower after WBC than pre-intervention, whereas testosterone/cortisol was significantly higher than pre-intervention (*p* < 0.05). The results of this study showed that a single session of WBC had a positive effect on accelerating the elimination of blood lactate after HIT, but did not significantly change rowing performance and physiological parameters. A single session of WBC was not an effective strategy for elite rowers for acute recovery after HIT.

## 1 Introduction

Rowers follow rigorous, carefully planned training programs designed to perform at their best in important competitions. High-intensity training (HIT) is inherent to the training programs of rowers and aims to apply a sufficiently high training pressure to further stimulate the body to improve athletic performance. Consequently, HIT may create an imbalance between training load and recovery of bodily functions, This can lead athletes into a state of overtraining, which ultimately leads to a decline in performance, immunity, and athleticism. Previous studies have demonstrated that HIT training for elite handball players increases hormonal and systemic inflammation throughout the body (Zwetsloot et al., 2014; Meckel et al., 2009). It has also been noted that failure to recover promptly after HIT may lead to symptoms such as increased fatigue, adverse mood changes, and a sustained decline in athletic performance (Le Meur et al., 2013; Le Meur et al., 2014). However, if there is an appropriate recovery period, an “overshoot” effect occurs, whereby physiological responses compensate for the training-related load and the athlete can perform better (Schaal et al., 2015). Therefore, methods that accelerate physical recovery after HIT may play an important role in improving performance.

Currently, different body cooling methods such as massages, active recovery, complete rest, and medications, have been demonstrated to restore the body’s functional state. Massages can help reduce muscle soreness and improve blood circulation, typically requiring several hours to a day to see benefits (Best et al., 2008). Active recovery, which involves low-intensity exercises, can promote blood flow and muscle repair, with optimal effects often observed within 24–48 h (Dupuy et al., 2018; Huyghe et al., 2018). Full rest allows the body to recover naturally over a period of days, depending on the extent of fatigue and muscle damage (Kellmann et al., 2018; Grandou et al., 2020). Medications, such as anti-inflammatories, can provide quicker relief from pain and inflammation but should be used judiciously due to potential side effects (Schellack and Ncube, 2021).

Whole-body cryotherapy (WBC) presents several advantages over these traditional methods, in which the participant is continuously exposed for 3 min to a very low temperature and dry freezer or cryo chamber (whose temperature ranges between −110°C and −160°C) (Rose et al., 2017). It works by applying a short, high-intensity cold stimulus to the skin surface all over the body, thereby relieving pain, treating inflammation, as well as improving athletic performance, and facilitating the rehabilitation process (Rose et al., 2017; Bouzigon et al., 2016; Mourot, Cluzeau, and Regnard, 2007; Christmas et al., 2016). Lactate is a widely recognized biomarker for assessing exercise intensity and the efficacy of recovery strategies. Various recovery techniques, such as passive rest, active recovery, and cold water immersion, have been shown to facilitate lactate clearance to varying degrees (Menzies et al., 2010; Hohenauer et al., 2015; Dupuy et al., 2018). However, WBC stands out for its pronounced effect on reducing blood lactate levels, making it a particularly effective method for enhancing recovery following high-intensity exercise (Wiewelhove et al., 2019). Unlike other methods, WBC’s extreme cold exposure may accelerate the reduction of lactate levels more effectively, thus mitigating muscle fatigue and shortening recovery time. This enhanced lactate clearance is crucial for maintaining and improving athletic performance (Thomas et al., 2007). Furthermore, WBC can influence hormonal responses, including reductions in cortisol and improvements in testosterone levels, which are vital for recovery and muscle repair (Hausswirth et al., 2011a). A recent review found that WBC was able to have significant effects on the physiological and biochemical parameters of athletes (Banfi et al., 2010). These results included a reduction in the release of pro-inflammatory cytokines, modulation of antioxidant status to adapt to environmental changes, and a reduction in the positive effects on muscle enzymes associated with muscle damage, such as creatine kinase and lactate dehydrogenase. This intervention approach has shown a wide range of positive effects across different athletic disciplines, not limited to improvements in physiological and biochemical parameters. (Ziemann et al., 2012a) documented that elite tennis players who underwent WBC at −120°C twice a day for a total of 5 days had higher stroke accuracy compared to an untreated control group; Krueger observed that WBC significantly improved acute recovery of long-distance runners during HIT at appropriate temperatures (2015). Although WBC has shown positive effects across multiple sports disciplines, research on its effectiveness and applicability in the specific field of rowing may still be insufficient. Rowing is a high-intensity, endurance-demanding sport, and its physical demands and recovery processes may differ from those of other types of sports.

WBC presents several advantages over these traditional methods. WBC involves exposing the entire body to extremely low temperatures for a short period, which can provide uniform and deep cooling. It has been shown to reduce pro-inflammatory cytokines, modulate antioxidant status, and decrease muscle damage markers such as creatine kinase and lactate dehydrogenase (Rose et al., 2017). Importantly, WBC has been associated with significant reductions in blood lactate levels, which is crucial for enhancing recovery after high-intensity training (Wiewelhove et al., 2019). Elevated lactate levels post-exercise can contribute to muscle fatigue and delayed recovery; hence, effective lactate clearance is beneficial for athletic performance (Duffield et al., 2010). Furthermore, WBC can influence hormonal responses, including reductions in cortisol and improvements in testosterone levels, which are vital for recovery and muscle repair (Hausswirth et al., 2020).

Therefore, the purpose of this study was to investigate the effects of a single session of WBC (3 min at −110°C) on recovery from HIT in elite rowers. It was hypothesized that a single session of WBC after HIT would induce modifications of biochemical and hematological parameters, which could be beneficial for accelerating recovery in elite rowers.

## 2 Materials and methods

### 2.1 Participants

G*power software (3.0.1, Univ. Kiel, Kiel, Germany) was used for priori estimation of the sample size (*a priori* power analysis) based on the prior data (salivary testosterone concentrations changes in the WBC group and the control group pre-exercise and immediately, 2 h and 24 h post-exercise, *f* = 0.70) showed that the minimum sample size required when performing repeated measures two-way ANOVA was 6 (*α* = 0.05, *β* = 0.2) (Russell et al., 2017a). Therefore, twelve elite male rowers (age 19.1 ± 8.4 years, height 193.7 ± 4.8 cm, weight 87.8 ± 8.4 kg, years of training 5.7 ± 3.1 years, and the best race time for 2 km 6:33 ± 0:47 min) with a sports grade of national level or higher were recruited from the national aquatic training base with no history of cold therapy intervention. The participants were randomized into a WBC group (n = 6) and a control group (CON, n = 6). They were asked to avoid alcohol and caffeine for 24 h before the tests and to adhere to their usual diet throughout the testing period. They were prohibited from using any other recovery methods, including physical therapy, nonsteroidal anti-inflammatory drugs, etc. Before the experiment, the participants were informed about the purpose and procedures of the study and written informed consent was obtained from the team coaches and the Aquatic Programmes Centre. The participants all signed an informed consent form and the study was ethically certified by the Ethics Committee of Ningbo University (No. TY2023026).

### 2.2 Procedure

**FIGURE 1:**
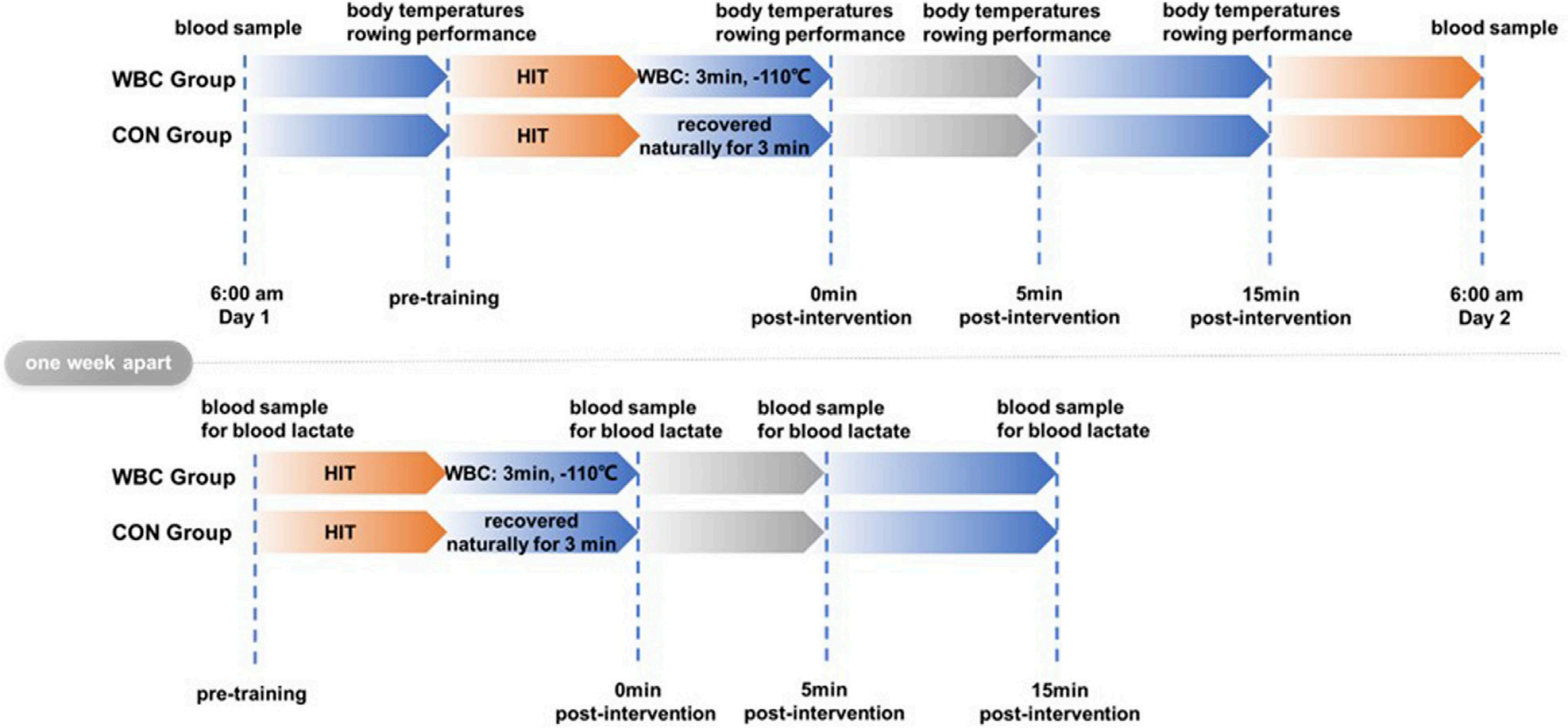
Experimental flow chart.

Each participant was asked to perform a 10 s maximal strength continuous rowing (Concept II RowErg, Concept II company, United States). This test was conducted both before (in a rested state) and immediately after a 1-day high-intensity training session (Table 1), as well as at the 5th and 15th minutes post-training (Lucertini et al., 2017). During the experiment, the participants were asked to complete 5 consecutive rows at maximal power to the best of their ability. Body temperatures (forehead, thighs, arms, and thighs), maximal power, energy expenditure, average speed, and segmentation time were recorded. The rowing team doctor cooperated in collecting venous blood samples (6 mL: 2 mL from the purple tube, and 4 mL from the red tube) from the elbows of the participants in the fasting state at 6:00 a.m. on the day of the high-intensity training and at the same time on the following day. To avoid the paddling test performed by the participants affecting the accuracy of the blood lactate collection, the blood lactate concentrations were therefore collected at the same time 1 week apart, specifically pre-HIT and immediately after the training session, at the 5th minute, and the 15th minute (Figure 1).

**TABLE 1:** High-intensity training program.

Time	Training protocol	Training intensity
8.00 9.30	Water training 26 km	Moderate
9.45 11.45	25 min of speed skating*4	High
14.00 15.10	Strength training	High
15.30 15.50	Running 12 laps (approximately 5 km)	Moderate

### 2.3 Intervention

After a day of HIT, the WBC group was subjected to cryotherapy intervention using a freezing chamber (Cryo°Cabin, Cryotech Nordic company, France). The chamber was pre-set to −110°C and continuously cooled for 300 s. Following the pre-cooling phase, the temperature was maintained at −110°C to keep it low temperatures. At the end of the training session, the participants in the WBC group were required to dry their bodies, remove all metal objects, and wear disposable cotton shoes and gloves. Subsequently, participants entered the cryotherapy chamber for 3 min of ultra-low temperature recovery at −110°C (Missmann et al., 2016). The height of the chamber was adjusted so that the shoulders aligned with its upper edge, leaving the area above the shoulders outside the chamber to prevent nitrogen gas inhalation. Throughout the intervention, participants’ heads were kept outside the chamber to avoid nitrogen inhalation, with a medically qualified doctor present to ensure safety and respond to emergencies. Additionally, the trial performer assessed participants’ wellbeing every minute and monitored changes in skin color and facial expression. A first aid kit was readily available in the experimental treatment room to address any potential hazards. The CON group was asked to recover naturally for 3 min.

The WBC intervention was repeated at the same time after a 1-week interval. The intervention protocol remained consistent with the procedures implemented during the initial week. Blood samples were collected in both groups from the earlobes for blood lactate testing in a resting state before the training, immediately, 5, and 15 min after the intervention, respectively.

### 2.4 Data processing

The slip distance (m), segment time (s), energy consumption (cal), maximum power (W), and average speed (m/s) were calculated using the concept II dynamometer (RowErg, Concept II, United States)). Beckman SYNCHRON CX^®^5 Pro automatic biochemical analyzer (Beckman, United States) was used to determine creatine kinase (CK). Beckman ACCESS^®^2 automatic chemiluminescent immunoassay analyzer (Beckman, United States) was used to determine white blood cell, red blood cell (RBC), hematocrit (HCT), cortisol (C), testosterone (T), cortisol/testosterone (T/C), hemoglobin (HGB). EKF glucose lactate analyzer (Biosen C-Line, Germany) was used to analyze blood lactate concentration in the participants’ earlobe blood. An electronic sphygmomanometer (HEM-7122, Omron, Japan) and thermometer (MC-720, Omron, Japan) were used to measure heart rate, diastolic blood pressure, systolic blood pressure, forehead temperature, arm temperature, and thigh temperature.

### 2.5 Statistical analysis

The data are presented as mean ± standard deviation (x ± s). All data were analyzed using SPSS statistical software (IBM SPSS v22.0, IBM Corp.). Shapiro-Wilk test was conducted to assess whether the data followed a normal distribution. A 2 × 4 repeated measure analysis of variance (ANOVA) was utilized to analyze the effects of different time points (pre-training, immediate post-intervention, 5 and 15 min post-intervention) on exercise recovery outcomes (rowing performance, skin temperature, blood pressure, and blood lactate concentration) within and between the two groups (WBC group versus CON group). A 2 × 2 repeated measure ANOVA was employed to analyze the biochemical indicators on both the morning of the training day and the morning after training. If ANOVA revealed a significant interaction or main effect, the Bonferroni test was used. Paired eta squared (*η* ^2^) was used as a measure of ANOVA effect size with 0.01, 0.06 and 0.14 considered small, medium and large, respectively. Cohen’s *d* was used as a measure of *post hoc* comparisons effect sizes with 0.2, 0.5 and 0.8 considered small, medium and large, respectively. Statistical significance was accepted at α = 0.05.

## 3 Results

### 3.1 Rowing performance

**FIGURE 2:**
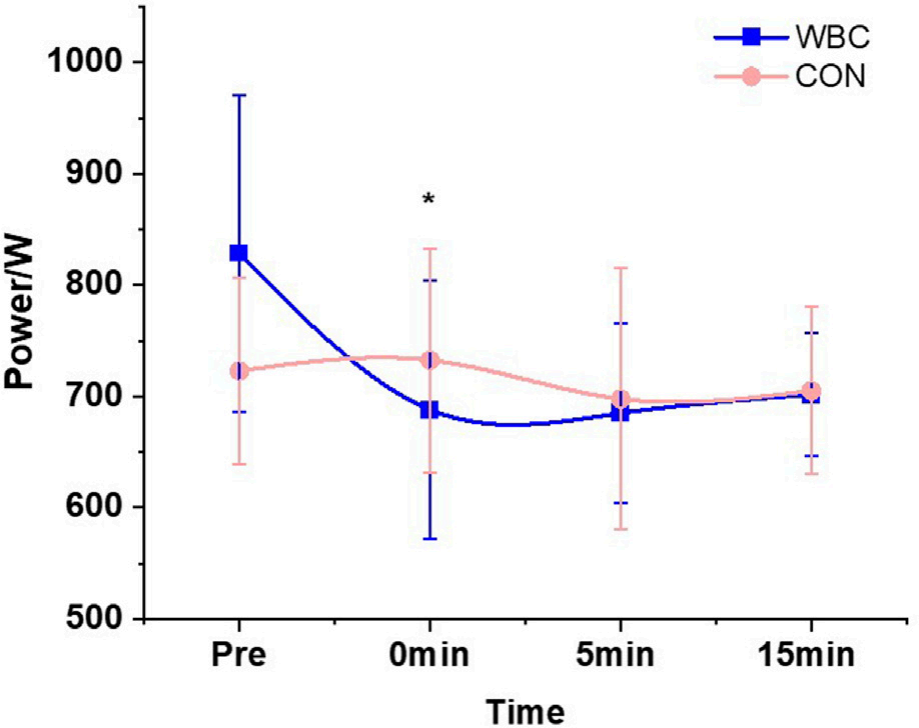
Effect of a single session of whole-body cryotherapy (WBC) on the maximum power during 10 s maximal strength continuous rowing performance (x ± SD). CON, control group. ^*^significant difference compared to the pre-training within the WBC group, *p* < 0.05.

The results showed that there was a significant interaction between group and time on the maximum power during 10s maximal strength continuous rowing (*p* < 0.05, *η* ^2^ = 0.262, Table 2). Post hoc analyses revealed that the maximum power in the WBC group significantly decreased immediately after the intervention compared to pre-training (*p* < 0.05, Cohen’s *d* = 0.475, Figure 2). A significant main effect of time was observed for average speed (*p* < 0.05), which significantly decreased immediately (Cohen’s *d* = 1.441), 5 (Cohen’s *d* = 1.169) and 15 min post-intervention (Cohen’s *d* = 1.134) compared to pre-intervention, and significantly decreased at 15 min post-intervention compared to at 5 min post-intervention (Cohen’s *d* = 0.909). No significant differences were found for the segmentation time, energy consumption, and glide distance (*p* > 0.05).

**TABLE 2:** Effect of a single session of whole-body cryotherapy (WBC) on 10s maximal strength continuous rowing performance (x±SD).

Variables	Groups	Pre-training	Immediately after WBC	5 min after WBC	15 min after WBC	*p*-value		
						Interaction (*η* ^2^)	Group (*η* ^2^)	Time (*η* ^2^)
MP (W)	WBC	828.5 ± 142.5	688.1 ± 116.1*	685.0 ± 80.7	701.6 ± 55.5	0.026 (0.262)	0.825 (0.005)	0.008 (0.322)
	CON	722.8 ± 84.1	732.5 ± 100.5	697.8 ± 117.5	705.3 ± 75.1			
ST (s)	WBC	93.6 ± 5.0	96.1 ± 4.2	96.1 ± 4.9	96.8 ± 3.4	0.696 (0.156)	0.788 (0.008)	0.599 (0.198)
	CON	94.8 ± 4.1	95.6 ± 3.9	95.0 ± 4.8	98.8 ± 3.3			
EC (cal)	WBC	8.1 ± 1.1	7.5 ± 1.0	7.3 ± 1.1	7.3 ± 0.8	0.082 (0.548)	0.693 (0.016)	0.312 (0.344)
	CON	7.3 ± 1.0	7.1 ± 0.4	7.8 ± 1.1	7.3 ± 0.5			
GD (m)	WBC	90.0 ± 4.6	88.0 ± 3.3	88.0 ± 7.0	85.0 ± 2.6	0.173 (0.445)	0.970 (0.001)	0.822 (0.102)
	CON	86.8 ± 3.3	87.4 ± 3.0	87.1 ± 4.6	88.8 ± 6.4			
AS (m/s)	WBC	54.5 ± 5.8	47.2 ± 4.2^#^	48.0 ± 5.3^#^	44.0 ± 3.0^#†^	0.406 (0.291)	0.793 (0.007)	0.030 (0.653)
	CON	51.3 ± 6.0	46.6 ± 4.8^#^	47.8 ± 4.1^#^	45.1 ± 3.0^#†^			

CON, control group; MP, maximum power; ST, segmentation time; EC, energy consumption; GD, glide distance; AS, average speed; *significant interaction between group and time, the difference compared to the pre-training, *p* < 0.05; ^#^significant main effect of time, the difference compared to the pre-training, *p* < 0.05; ^†^significant main effect of time, the difference compared to 15 min after WBC, *p* < 0.05.

### 3.2 Skin temperature, heart rate, and blood pressure

**FIGURE 3:**
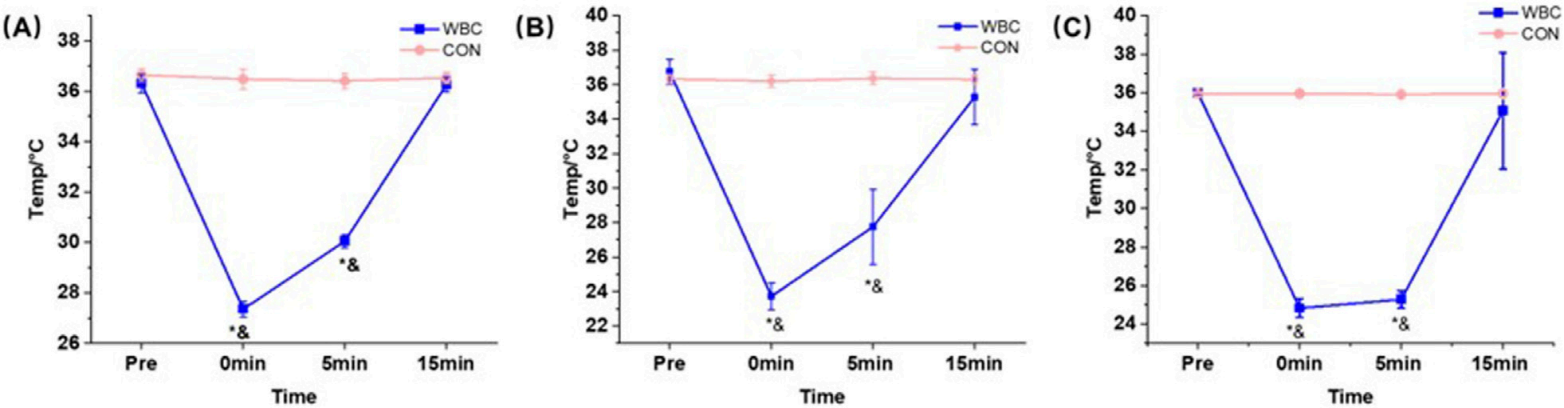
Effect of a single session of whole-body cryotherapy (WBC) on the forehead **(A)**, upper arm **(B)**, and thigh **(C)** temperatures (x ± SD); CON, control group. ^*^significant difference within the WBC group compared to the pre-training group, *p* < 0.05; ^&^significant difference between the two groups at the same test time, *p* < 0.05.

The results demonstrated a significant interaction between group and time on the temperatures of the forehead (*p* < 0.05, *η* ^2^ = 0.413, Table 3), upper arm (*p* < 0.05, *η* ^2^ = 0.832, Table 3), and thigh (*p* < 0.05, *η* ^2^ = 0.879, Table 3). Post hoc analyses revealed that the temperatures in the WBC group were significantly lower than those during the pre-training immediately after the intervention (Cohen’s *d*_forehead_ = 0.943, Cohen’s *d*_upper arm_ = 0.892, Cohen’s *d*_thigh_ = 1.256) and at the 5 min post-intervention (Cohen’s *d*_forehead_ = 0.937, Cohen’s *d*_upper arm_ = 1.093, Cohen’s *d*_thigh_ = 0.846), and were also significantly lower compared to the CON group (*p* < 0.05, Cohen’s *d*_forehead_ = 1.198, Cohen’s *d*_upper arm_ = 0.931, Cohen’s *d*_thigh_ = 1.249, Figure 3). However, heart rate and blood pressure indicators were not significantly different before and after the intervention (*p* > 0.05).

**TABLE 3:** Effect of a single session of whole-body cryotherapy (WBC) on the skin temperature, heart rate, and blood pressure (x ± SD).

Variables	Groups	Pre-training	Immediately after WBC	5 min after WBC	15 min after WBC	*p*-value		
						Interaction (η^2^)	Group (η^2^)	Time (η^2^)
PR (times/min)	WBC	72.0 ± 12.9	78.3 ± 14.9	75.3 ± 18.6	75.3 ± 18.6	0.103 (0.519)	0.836 (0.004)	0.166 (0.452)
	CON	76.5 ± 9.1	70.3 ± 5.5	73.8 ± 9.9	69.7 ± 11.8			
SBP (mmHg)	WBC	125.1 ± 6.1	130.0 ± 6.8	131.3 ± 11.0	124.5 ± 6.4	0.433 (0.272)	0.208 (0.153)	0.563 (0.215)
	CON	134.1 ± 5.8	127.5 ± 8.5	129.6 ± 6.5	129.5 ± 5.2			
DBP (mmHg)	WBC	56.1 ± 5.5	61.3 ± 6.9	72.5 ± 13.5	60.8 ± 6.2	0.053 (0.597)	0.339 (0.091)	0.703 (0.153)
	CON	72.0 ± 21.6	59.3 ± 5.8	52.5 ± 5.2	57.1 ± 6.5			
Forehead temperature (°C)	WBC	36.3 ± 0.3	27.3 ± 0.3^*&^	30.0 ± 0.2^*&^	36.2 ± 0.3	0.036 (0.413)	0.021 (0.445)	0.015 (0.549)
	CON	36.6 ± 0.2	36.4 ± 0.3	36.4 ± 0.2	36.5 ± 0.2			
Upper arm temperature (°C)	WBC	36.7 ± 0.7	23.7 ± 0.7^*&^	27.7 ± 2.1^*&^	35.2 ± 1.6	<0.01 (0.832)	<0.01 (0.886)	<0.01 (0.914)
	CON	36.3 ± 0.2	36.2 ± 0.3	36.3 ± 0.3	36.3 ± 0.2			
Thigh temperature (°C)	WBC	35.9 ± 0.1	24.8 ± 0.4^*&^	25.2 ± 0.4^*&^	35.0 ± 3.0	<0.01 (0.879)	<0.01 (0.913)	<0.01 (0.878)
	CON	35.9 ± 0.1	35.9 ± 0.1	35.9 ± 0.0	35.9 ± 0.1			

CON, control group. *significant difference within the WBC group, compared to the pre-training group, *p* < 0.05; ^&^significant difference between the two groups at the same test time, *p* < 0.05; PR, pulse rate; SBP, systolic blood pressure; DBP, diastolic blood pressure.

### 3.3 Blood parameters

**FIGURE 4:**
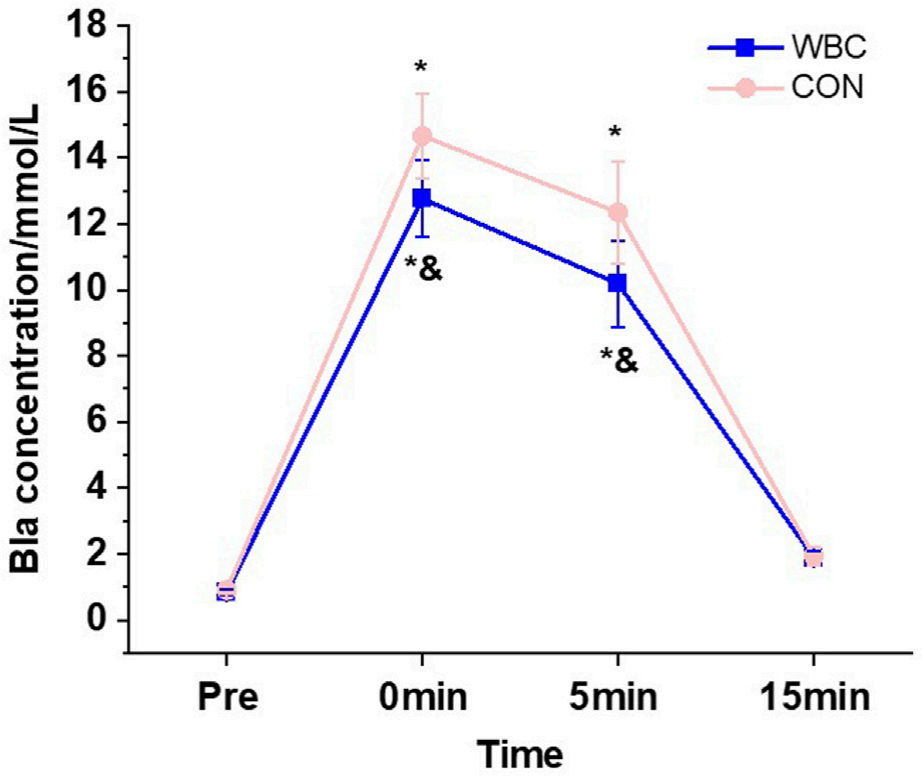
Effect of a single session of whole-body cryotherapy (WBC) on blood lactate concentration; CON, control group. ^*^Significant difference compared to pre-training, *p* < 0.05; ^&^significant difference between the two groups at the same test time, *p* < 0.05.

The results of the study showed a significant interaction effect of group and time on blood lactate concentration (*p* < 0.05, Table 4). Post hoc analysis revealed that blood lactate concentrations in the WBC group immediately (Cohen’s *d* = 1.158) and 5 min (Cohen’s *d* = 1.365) after WBC were significantly lower than pre-training and significantly lower than the CON group (Cohen’s *d* = 0.877, Figure 4); the blood lactate concentration in the WBC group at 15 min after WBC was significantly higher than pre-training (Cohen’s *d* = 1.214), but did not differ significantly from the CON group (*p* > 0.05).

**TABLE 4:** Effect of a single session of whole-body cryotherapy (WBC) on blood lactate concentration (x±SD).

Groups	Pre-training (mmol/L)	Immediately after WBC (mmol/L)	5 min after WBC (mmol/L)	15 min after WBC (mmol/L)	*p*-value		
					Interaction (*η* ^2^)	Group (*η* ^2^)	Time (*η* ^2^)
WBC	0.9 ± 0.1	12.8 ± 1.2^*&^	10.2 ± 1.3^*&^	1.9 ± 0.2^*^	0.013 (0.745)	0.025 (0.532)	<0.01 (0.857)
CON	0.9 ± 0.1	14.7 ± 1.3^*^	12.4 ± 1.6^*^	1.9 ± 0.3^*^			

CON, control group. *significant difference compared to pre-training, *p* < 0.05; ^&^significant difference between the two groups at the same test time, *p* < 0.05.

**FIGURE 5:**
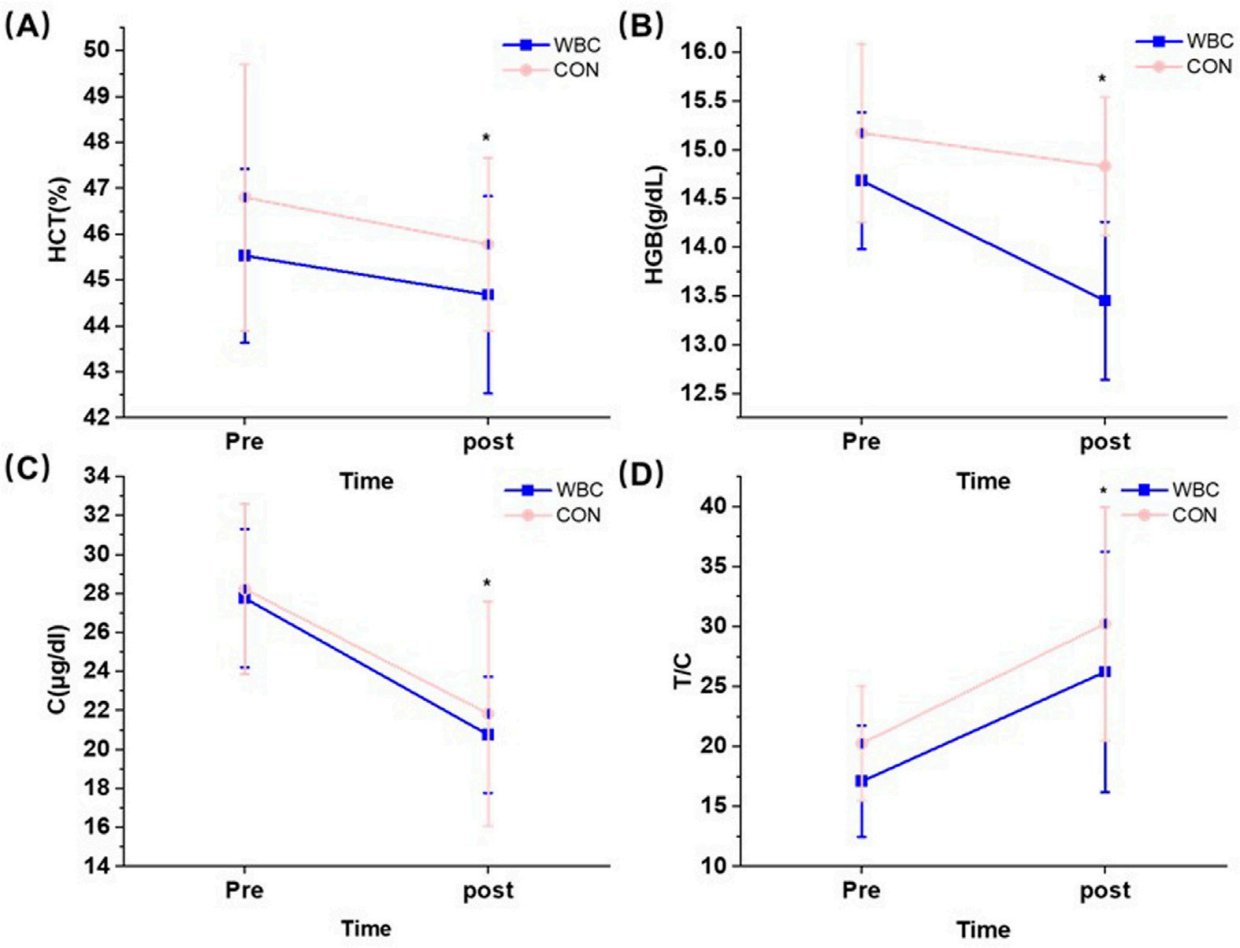
Effect of a single session of whole-body cryotherapy (WBC) on HCT **(A)**, HGB **(B)**, C **(C)**, and T/C **(D)** (x ± SD); HCT, hematocrit; HGB, hemoglobin; C, cortisol; T/C testosterone/cortisol; CON, control group; ^*^significant difference compared to pre-training, *p* < 0.05.

The results showed that there was no interaction for all biochemical indices (*p* > 0.05), but the significant main effect of time was observed (*p* < 0.05, Table 5). Specifically, HCT (*η* ^2^ = 0.325), C (*η* ^2^ = 0.673), and HGB (*η* ^2^ = 0.396) were significantly lower after the intervention than pre-intervention (*p* < 0.05) whereas T/C was significantly higher than pre-intervention (*η* ^2^ = 0.551, Figure 5).

**TABLE 5:** Effect of a single session of whole-body cryotherapy (WBC) on biochemical indicators (x±SD).

Variables	Groups	Pre	Post	*p*-value		
				Interaction (*η* ^2^)	Groups (*η* ^2^)	Times (*η* ^2^)
RBC (10^3/uL)	WBC	5.1 ± 0.2	5.0 ± 0.1	0.896 (0.002)	0.363 (0.083)	0.170 (0.229)
	CON	5.2 ± 0.2	5.1 ± 0.1			
HCT (%)	WBC	45.5 ± 1.9	44.6 ± 2.1	0.849 (0.004)	0.363 (0.083)	0.048 (0.325)
	CON	46.8 ± 2.9	45.7 ± 1.8			
WBC^1^ (10^3/uL)	WBC	5.8 ± 0.7	5.8 ± 0.8	0.370 (0.081)	0.952 (0.001)	0.160 (0.174)
	CON	6.0 ± 0.6	5.7 ± 0.9			
CK (U/L)	WBC	544.8 ± 234.9	404.6 ± 139.3	0.174 (0.176)	0.815 (0.006)	0.477 (0.052)
	CON	509.0 ± 280.2	580.6 ± 405.9			
C (μg/dL)	WBC	27.7 ± 3.5	20.7 ± 2.9	0.839 (0.004)	0.706 (0.015)	0.012 (0.673)
	CON	28.2 ± 4.3	21.8 ± 5.7			
T (ng/dL)	WBC	467.1 ± 121.5	520.0 ± 135.9	0.942 (0.001)	0.151 (0.195)	0.139 (0.206)
	CON	561.0 ± 117.9	619.0 ± 114.5			
T/C	WBC	17.0 ± 4.6	26.2 ± 10.0	0.878 (0.002)	0.359 (0.085)	0.006 (0.551)
	CON	20.2 ± 4.7	30.2 ± 9.7			
HGB (g/dL)	WBC	14.6 ± 0.7	13.4 ± 0.8	0.661 (0.020)	0.350 (0.088)	0.028 (0.396)
	CON	15.1 ± 0.9	14.8 ± 0.7			

WBC^1^, white blood cell; RBC, red blood cell; HCT, hematocrit; CK, creatine kinase; C, cortisol; T, testosterone; T/C, testosterone/cortisol; HGB, hemoglobin; WBC, Whole-Body Cryotherapy group; CON, control group.

## 4 Discussion

The current study aimed to investigate the effects of WBC (3 min at −110°C) on acute recovery after HIT in elite rowers. None of the athletes reported any injuries or negative side effects related to the cold exposure, which indicated a single session of WBC applied in the current study, may be administered without health risk in elite athletes. The main finding of this study was that the blood lactate concentrations and the maximum power decreased significantly in the WBC group after a single session of WBC. However, there were no significant effects on other blood parameters and exercise performance.

The results of this study highlighted the impact of WBC on blood lactate concentration recovery post-exercise. Specifically, the blood lactate concentrations in the WBC group immediately and 5 min after WBC were 87.1% and 82.5% of those in the CON group at the same moment, respectively. These results indicated that the recovery of blood lactate in the WBC group was significantly faster than that in the CON group, which proved the facilitating role of WBC on the rapid recovery of blood lactate levels in rowers after high-intensity training. This result was similar to that of Wilson and Shepler. (2017) who explored the difference in blood lactate levels between the sedentary and WBC (−120°C, 3°min) groups among Brazilian jiu-jitsu athletes after strenuous exercise. Their study revealed that the WBC group had significantly lower blood lactate concentrations than the sedentary group at both the 10th and 15th minutes post-exercise. Similarly, (Lee and Jeong, 2003) assessed the differences in the rate of blood lactate recovery among seven athletes following a 400 m run through the ice and passive recovery methods. The findings indicated that the blood lactate concentration during the recovery phase was significantly lower in the ice recovery group than that of the passive recovery group in the 5th minute of the recovery period. It has been demonstrated that as a metabolic product of glycolysis for energy during exercise, lactate is produced in large amounts after intense exercise (Hoff et al., 2016). After WBC, the temperature of each part of the participants decreased rapidly, and this study showed that the temperature of the forehead, thighs, and arms decreased by an average of 8.95°C, 13°C, and 11.15°C, respectively, immediately after WBC. Vasodilatation induced by the cold stimulus may be the main reason for the faster rate of blood lactate decrease, and studies have demonstrated that the blood flow is four times higher than normal after WBC and that this rapid blood circulation change can last for hours after exercise and blood lactate metabolism is also more rapid (Bouzigon et al., 2021). Another possibility was that WBC could accelerate functional recovery by reducing muscle catabolic activity thereby reducing blood lactate production (Patel et al., 2019). It is therefore recommended for athletes to integrate WBC into their post-intensive training regimen to enhance recovery processes.

Although this study observed an accelerated clearance of blood lactate after WBC, this effect did not translate into alterations in segmentation time, skating distance, or energy expenditure. The results revealed no significant differences between groups in these parameters, except for a significant reduction in immediate post-cold therapy maximal power in the WBC group, compared to pre-training levels. Similar to the results of the present study, Russell et al. (2017b) indicated a single session of WBC (−135°, 3 min) had no enhancement effect on immediate short-distance sprinting (15*30 m) in football players, except for elevating testosterone concentrations over 24 h. In addition, Ferreira et al. (2014) found peak maximal elbow flexion moments were reduced 10 min after a single session of WBC (−110°, 3 min) on strong, healthy young men compared to the pre-test. Furthermore, Fullam et al. (2015) found that a single 15-min cryotherapy session on the ankle joints had a negative postural balance. These studies, aligning with the present research, suggested that while WBC might offer certain benefits, such as elevated testosterone levels, its impacts on immediate athletic performance and muscular function can vary, sometimes resulting in no improvement or even negative outcomes. Conversely, other research offered a different perspective on the potential benefits of WBC. Krueger et al. (2015) observed that WBC significantly improved acute recovery during high-intensity exercise at −110°C for 3 min for long-distance runners; (Ziemann et al., 2012b) documented improvements in athletic performance in a group of outstanding tennis players after WBC. They found that athletes who underwent WBC at −120°C twice a day for a total of 5 days had higher stroke accuracy compared to untreated controls. These findings indicated that there were differences in the effectiveness of WBC in enhancing athletic performance and muscle function, highlighting the complexity of the effects of WBC interventions.

There was only a significant main effect of time in the blood parameters, with no significant differences between the groups. Specifically, HCT, C, and HGB were significantly lower after the intervention than pre-intervention, whereas T/C was significantly higher than pre-intervention. This result was consistent with previous studies on a single session of WBC. Jurecka et al. (2023) assessed the effect of a single session of WBC (−130°C, 3°min) on professional runners and showed no significant differences in CK and inflammatory reactants (e.g., white blood cell) after a single session of WBC. A similar result was obtained in the study of Krueger et al. (2015), who found that a single session of WBC did not have a significant effect on leukocytes, C, T, and CK after high-intensity intermittent running exercise. However, it was notable that while acute WBC sessions might not sufficiently impact biochemical indices, repeated exposure could induce significant physiological changes. Banfi et al. (2009) demonstrated that rugby players who underwent a WBC once a day during 5°days of training (−110°C, 3°min) had no effect on their immune parameters but showed a decrease in pro-inflammatory cytokines versus an increase in anti-inflammatory cytokines. Ptaszek et al. (2023). Measured the effect of prolonged WBC (−120°C, 3 min) on blood morphology and rheological parameters in healthy participants and showed that WBC resulted in significant decreases in RBC, HGB, HCT, and positively affected blood rheology. We speculated that this might be due to the cumulative effects of WBC on the recovery mechanisms. A single session of WBC may not produce a strong enough stimulus to elicit marked changes in the biochemical and inflammatory responses. However, repeated sessions of WBC could introduce increased stimulus or load, resulting in enhanced anti-inflammatory responses and blood rheology. This increased stimulus might contribute to enhanced recovery processes, highlighting the importance of considering the duration and frequency of WBC in athletic training regimens. Another potential reason was the exercise intensity before WBC. The participants involved in our study were elite-level rowers, who have adapted to the intensity and cold stress of winter training over a long period. Consequently, reliance on a solitary session of WBC might not be sufficient to cause significant changes in blood indices. This might explain the inconsistency between the present study and that of Pournot et al. (2011) who found significantly lower inflammatory markers in the blood of professional runners after a single session of WBC following simulated cross-country running. This significant observation could be attributed to the unique physiological challenges posed by cross-country running. Specifically, the heavy loads characteristic of this type of running were known to induce substantial muscle structural damage and inflammation, potentially explaining the efficacy of a single session of WBC session in their study (Hausswirth et al., 2011b). Therefore, these findings underscored the importance of considering the intensity of the pre-exercise load as well as the frequency and duration of WBC when evaluating it as a strategy for post-exercise recovery. Lastly, an important aspect that may have affected the results of the trial was the relatively small size of the experimental group. This study should be continued in larger groups.

There are some limitations of this study. Firstly, since we specifically focused on male athletes, female athletes were not included in the study. Females have been indicated to cool down faster than males after WBC (Cuttell et al., 2017), thus it is reasonable to speculate that there are differences in athletic performance, and physiological and biochemical parameters between genders after WBC. Secondly, the participants in this study were all elite athletes who had adopted HIT. The recovery effect of a single session of WBC may be limited for elite athletes. Therefore, it is recommended that future studies include female athletes and conduct long-term investigations to make the research more comprehensive.

## 5 Conclusion

The results of this study showed that a single session of WBC had a positive effect on accelerating the elimination of blood lactate after HIT. However, the intervention did not significantly change rowing performance and physiological parameters. A single session of WBC was not an effective strategy for elite rowers for acute recovery after HIT.

## Data Availability

The original contributions presented in the study are included in the article/supplementary material, further inquiries can be directed to the corresponding author.
